# Effects of Nicotine on Emotional Reactivity in PTSD and Non-PTSD Smokers: Results of a Pilot fMRI Study

**DOI:** 10.1155/2012/265724

**Published:** 2012-06-03

**Authors:** Brett Froeliger, Jean Crowell Beckham, Michelle Feldman Dennis, Rachel Victoria Kozink, Francis Joseph McClernon

**Affiliations:** ^1^Department of Psychiatry and Behavioral Sciences, Duke University Medical Center, Durham, NC 27708, USA; ^2^Durham Veterans Affairs Medical Center, Durham, NC 27708, USA; ^3^VISN 6, Mental Illness Research, Education, and Clinical Center (MIRECC), Durham, NC 27708, USA

## Abstract

There is evidence that individuals with posttraumatic stress disorder (PTSD) may smoke in part to regulate negative affect. This pilot fMRI study examined the effects of nicotine on emotional information processing in smokers with and without PTSD. Across groups, nicotine increased brain activation in response to fearful/angry faces (compared to neutral faces) in ventral caudate. Patch x Group interactions were observed in brain regions involved in emotional and facial feature processing. These preliminary findings suggest that nicotine differentially modulates negative information processing in PTSD and non-PTSD smokers.

## 1. Introduction

Posttraumatic stress disorder (PTSD) is associated with elevated rates of cigarette smoking (40%–63%) compared with population norms (20%–30%) [1–3]. Moreover, smokers with PTSD are significantly more likely to be “heavy” smokers (i.e., smoke >25 cigarettes/day) [4] and take larger puffs [5]. In naturalistic studies, PTSD smokers are more likely to report negative affective (NA) states as an antecedent to smoking [6] and also report significant reductions in NA following smoking [7].

A hallmark phenotype of individuals with PTSD is increased psychophysiological responsivity and NA to idiopathic trauma-related stimuli [8]. Furthermore, individuals with PTSD exhibit aberrant responding to nonspecific, negative emotional stimuli [9]. For instance, individuals with PTSD exhibit biased attention to negative emotional information [10, 11]. Moreover, compared to non-PTSD trauma survivors, PTSD survivors have increased electrocortical responses to sad faces [12]. It has been proposed [13, 14] that dysregulated emotional information processing in PTSD is due to hyperresponsiveness of the amygdala—a region subserving negative emotional information processes [15]—and also hyporesponsiveness of medial prefrontal cortices—a region involved in cognitive control of emotional responses [16]. Support for this hypothesis comes from fMRI studies of PTSD patients showing increased reactivity to fearful faces in amygdala as compared to controls [17, 18] coincident with decreased reactivity in medial prefrontal regions [18].

Laboratory studies show that smoking and nicotine reduces distraction caused by negative stimuli [19] and electrocortical responses [20] to these stimuli among smokers. Moreover, neuroimaging studies show that nicotine acts on limbic (e.g., amygdala) and prefrontal brain areas that subserve emotional information processing [21–23]. Despite evidence regarding smoking/PTSD interactions, no neuroimaging studies to date have evaluated the neurobiological basis of nicotine and/or smoking effects on emotional information processing among individuals with PTSD. Thus, we conducted a preliminary study aimed at evaluating this question. Smokers with and without a PTSD diagnosis underwent fMRI scanning 2 hrs after application of a 21 mg transdermal nicotine or placebo patch. During scanning participants viewed emotional or neutral face stimuli. We hypothesized that nicotine and PTSD, both separately and in combination, would have effects on brain activation, specifically in regions underlying emotional processes.

## 2. Materials and Methods

### 2.1. Subjects and Stimuli

Participants (*n* = 11) were adult smokers with and without PTSD recruited from community and clinic sources. Eligibility requirements included being between the ages of 18–75, smoking ≥10 cigarettes per day over the past year, abstinence from nicotine delivery other than cigarettes, having 20/20 corrected vision, native English speaking, free of any neurological history, or major medical problems, passing a urine drug screen and pregnancy test if female and not meeting DSM-IV criteria for current drug or alcohol abuse/dependence (except nicotine). Participants read and signed an Institutional Review Board approved informed consent form and were paid $250 upon study completion. Eleven participants completed all aspects of the study. Data from 3 participants were excluded due to computer hardware difficulty (*n* = 2) and data-related problems (*n* = 1).

### 2.2. Procedure

 Participants completed three sessions—one screening/diagnostic and two scanning sessions. PTSD diagnosis was based on the Clinician Administered PTSD Scale [24]. Other psychiatric disorders were diagnosed based on the Structured Clinical Interview for DSM-IV diagnosis [25]. Current alcohol and drug abuse/dependence diagnoses were determined by a 3-month time frame; current diagnoses for major depressive episode and anxiety disorders were determined by a 1-month time frame. Two trained raters (kappa for diagnoses =  .97) conducted the interviews under the supervision of a licensed clinical psychologist (JCB). The Beck Depression Inventory (BDI), the Fagerström test for Nicotine Dependence (FTND), and a smoking history form were administered (see Table 1).

 On each experimental day, participants were administered either a transdermal nicotine (21 mg NicoDerm) or placebo patch. Placebo patches (resembling nicotine patches) were manufactured by 1–800-PATCHES. Participants were instructed to smoke as usual up to patch administration. The patch was placed on the lower upper arm to avoid complications during scanning. In the 2 hrs following patch application, participants maintained smoking abstinence and were monitored by study personnel. After 2 hours, participants entered the MRI suite, were placed in the scanner, and then performed an experimental task during fMRI scanning. Patch order was randomly assigned and counterbalanced across participants.

### 2.3. Experimental Task

**Figure 1:**
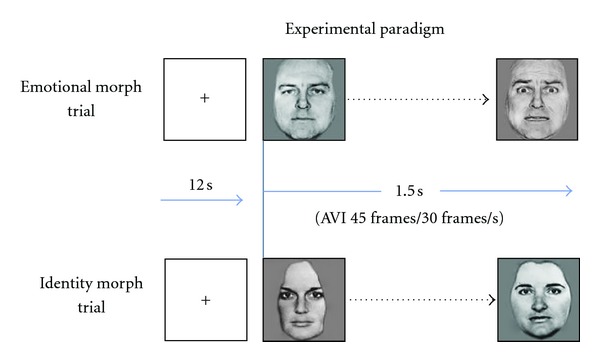
fMRI task paradigm.

The experimental task was a modified version of a face viewing task previously shown to increase activation in brain regions underlying emotion processing [26]; see Figure 1. In brief, neutral and negative (angry and fearful) faces [27] were presented in a dynamic (i.e., morphed) fashion. The morphing caused them to appear to change from neutral to negative in the same actor (emotion morph) or from one neutral identity to another neutral identity (identity morph). Trials were separated by a fixation cross. Participants used a response box to indicate whether each face depicted an emotion or an identity morph. Stimuli were presented in a pseudorandom event-related design. The intertrial interval varied between 12 and 15 s (M = 13.5 s). Each session was divided into eight, 8 min 24 s runs. Run order was counterbalanced across participants.

### 2.4. Scanning Procedures

MR images were acquired on a 1.5 T General Electric Signa NVi scanner (Milwaukee, WI, USA) equipped with 41 mT/m gradients. The participant's head was immobilized using a cushion and tape. The anterior and posterior commissures were identified in the midsagittal slice of a localizer series. A high-resolution T1-weight anatomical image was then acquired (124 contiguous slices, repetition time, TR = 8.2 s, TE = 3.3 ms, FOV = 24 cm, matrix = 256^2^, slice thickness = 1.5 mm). Functional images were collected during the task with an inverse spiral pulse sequence sensitive to blood-oxygenation-level-dependent (BOLD) contrast (30 slices, TR = 1.5 s, TE = 10 ms, FOV = 24 cm, matrix = 64^2^, flip angle = 81°, slice thickness = 3.8 mm, in-plane resolution = 3.75 mm^2^).

### 2.5. Data Analysis

The fMRI data analysis utilized a voxel-based approach implemented in SPM5 (Wellcome Trust Centre for Neuroimaging, London, UK). Preprocessing steps included (1) slice-time correction, (2) realignment using rigid body translation and rotation, (3) normalization into a standard stereotaxic space (Montreal Neurological Institute) with an isotropic 2 mm^3^ voxel size, and (4) smoothing with an 8 mm Gaussian filter.

For each participant on each session, statistical parametric maps were derived by applying linear contrasts to the parameter estimates for the event of interest (emotional morph > identity morph), resulting in a *t*-statistic for every voxel. These contrasts were then passed onto the second level for random-effects analyses. Statistical contrasts were set up to calculate signal differences between patch condition (nicotine versus placebo), group (PTSD versus control), and the 2-way interaction between patch and group. A gray matter mask was applied to statistical parametric maps, and results were thresholded at *P* < 0.001, uncorrected, with a spatial extent of ten contiguous voxels.

## 3. Results

### 3.1. Participant Demographics

Participants were adult smokers with (*n* = 4) and without PTSD (*n* = 4). See Table 1 for smoking history and demographic information. Groups were matched on age (sample M = 36.6, SD = 14.2), average number of years smoked (M = 20, SD = 14.3), cigarettes per day (M = 19.2, SD = 6.8), and nicotine dependence (FTND score M = 5.5; SD = 2.4).

### 3.2. fMRI Activations

**Figure 2:**
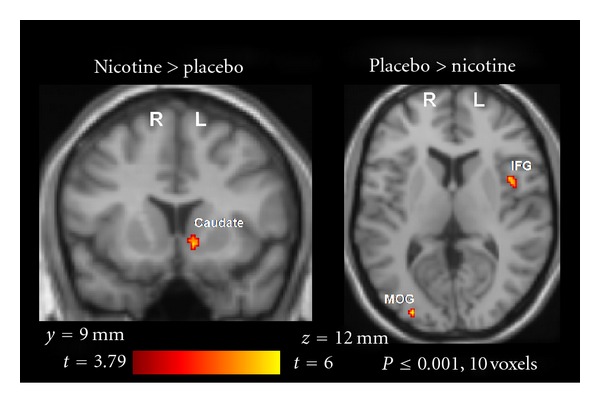
fMRI contrast of the main effects of patch type. Across groups, activation (emotion morph > identity morph) was greater for nicotine versus placebo patch in left caudate (*x* = −10, *y* = 14, *z* = −6), whereas greater activation was observed in left inferior frontal gyrus (IFG) (*x* = −42, *y* = 8, *z* = 8) and right middle occipital gyrus (MOG) (*x* = 32, *y* = −88, *z* = 4) for placebo versus nicotine patch.

**Figure 3:**
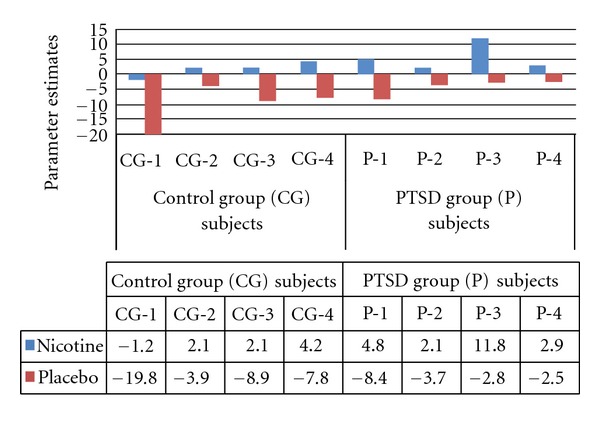
Model parameter estimates of the main effect of patch type on task-related left caudate (*x* = −10, *y* = 14, *z* = −6) BOLD response.

*Patch effects* Across groups, nicotine patch compared to placebo resulted in increased activation in left ventral caudate (Figures 2 and 3). As represented in Figure 2, significant activations for placebo relative to nicotine patch were observed in right middle occipital gyrus (BA 19) and left inferior frontal gyrus (BA 44).

**Group Effects**

Across patch conditions, activation was significantly greater in the PTSD as compared to non-PTSD group in striatum, amygdala, and frontal, parietal, and occipital cortices (see Table 2). No activations were greater in the non-PTSD relative to PTSD group.

**Patch x Group Interaction**

**Figure 4:**
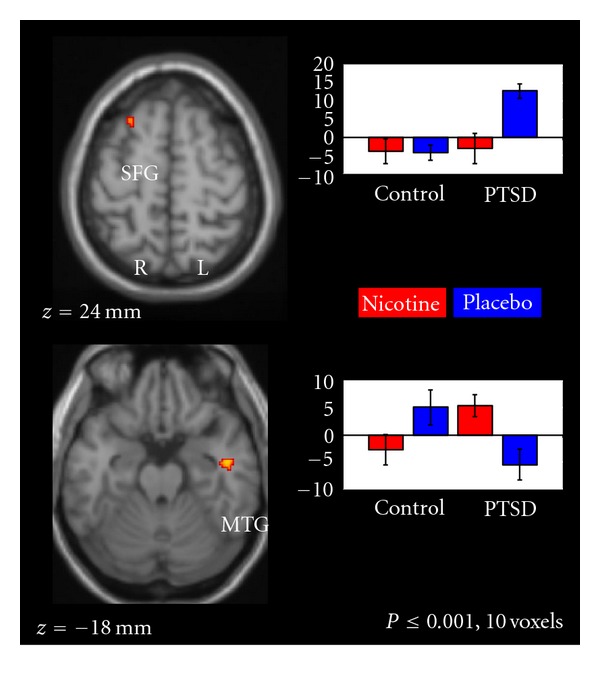
fMRI contrast of the patch x group interactions. Significant patch x group interactions were observed in right superior frontal gyrus (SFG) (*x* = 24, *y* = 22, *z* = 60) and left middle temporal gyrus (MTG) (*x* = −48, *y* = −10, *z* = −24).

**Figure 5:**
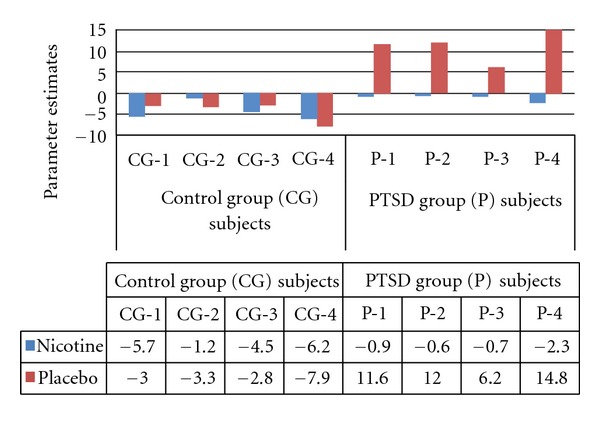
Model parameter estimates of the group x patch interaction on task-related right superior frontal gyrus (SFG) (*x* = 24, *y* = 22, *z* = 60) BOLD response.

As represented in Figure 4, patch x group interactions were observed in right superior frontal gyrus (SFG) (Figure 5) and left middle temporal gyrus (MTG). In SFG, activation was greatest in the PTSD group than that in the placebo condition. In MTG, activation was greater in the PTSD group in the nicotine relative to placebo condition; the opposite pattern was observed in the non-PTSD group.

## 4. Discussion

This preliminary study is the first to systematically assess the effects of nicotine on neural correlates of emotional information processing in a PTSD sample. As in previous studies [17, 18], PTSD was associated with larger brain responses to emotional face stimuli in amygdala and prefrontal regions.

In evaluating the effects of nicotine, we observed patch x group interactions in several brain areas which suggest nicotine might modulate emotional information processing *via *different neural mechanisms in smokers with and without PTSD. The observed patch x group interaction in SFG suggested greater reactivity to emotional cues in this region when smokers with PTSD were in a nicotine-deprived state. The SFG plays an important role in emotion, memory, and motivational processes. As compared to controls, individuals with PTSD have been shown to exhibit increased activation in SFG upon recall of neutral information that was encoded in an emotional context [28]. A patch x group interaction was also observed in MTG—a region previously shown to be selectively active in response to nonaffective components of face stimuli (e.g., perception and familiarity; [29]). This area was more reactive to emotional face cues in smokers with PTSD when receiving nicotine. Collectively, these findings suggest that nicotine (and nicotine deprivation) may modulate reactivity to the nonaffective and affective components of face stimuli in smokers with PTSD.

In addition to the above interactions, we observed a main effect of nicotine in which activation to emotional face stimuli was greater in left ventral caudate following nicotine patch administration. The ventral caudate is part of the ventral striatum—a brain region that mediates reward processes [30]. Nicotine stimulates the release of dopamine in the ventral striatum in both animals [31] and in human smokers [32]. Likewise, nicotine abstinence results in decreased ventral striatal dopamine functioning [33]. Thus, our novel findings of nicotine-induced increases in reactivity to emotional stimuli in the striatum may be due to increased dopamine transmission in this region brought on by nicotine administration (or decreases in dopamine neurotransmission in the absence of nicotine).

The present study has limitations including a small sample size and a relatively heterogeneous sample with respect to age, psychiatric comorbidity, and smoking history. We manipulated nicotine in the context of brief abstinence so it remains unknown what effect a longer abstinence period would have on emotional information processing. Additional work with larger samples and under other clinically relevant conditions is needed.

## 5. Conclusion

The present preliminary study provides novel information regarding the effects of nicotine on emotional information processing in smokers with and without PTSD. Smokers with PTSD report greater NA immediately prior to smoking [34] and greater decreases in NA following smoking [35], and these findings are consistent with the observed patterns of brain activation in the current study. Thus, our findings provide a neurobiological basis that helps explain why individuals with PTSD are at greater risk of smoking and also experience greater difficulty quitting. The present study is not without its limitations. Our sample size was small and was predominately represented by female smokers. Moreover, among the female participants, we did not obtain information regarding menstrual cycle phase in relation to the timing of each of their experimental sessions which may have added some variance to the results. Future work will examine the effects of nicotine and smoking in larger samples of smokers with PTSD, control for sex differences, and among females control for time in menstrual cycle, and relate these findings to smoking-related outcomes (e.g., smoking cessation success/failure).

## Figures and Tables

**Table 1:** Participant demographics^ab^.

Sub.	Age	Sex	FTND	Cigs/day	Yrs smoked	BDI	PTSD(CAPS)	Psychiatric history	Trauma event	Drug dep. hx
P-1	27	F	5	10	9	1	Yes	MDD, OCD, Adjustment disorder	Witness to assault	Alcohol
P-2	41	F	3	20	25	11	Yes	None	Death of daughter	None
P-3	28	F	8	50	11	24	Yes	Agoraphobia, specific phobia (heights), MDD	Children removed by social services	None
P-4	31	F	6	15	15	9	Yes	MDD	Child sexual abuse	
C-1	68	F	5	60	49	2	No	MDD	Death of husband	Alcohol
C-2	27	M	4	15	8	0	No	None	Hurricane	None
C-3	28	F	3	13	12	1	No	None	None	None
C-4	43	F	10	39	31	10	No	Subthreshold OCD	Death of friend	None

^a^PTSD group subjects are denoted as de-identified subject numbers P-1 through P-4.

^b^Control group subjects are denoted as de-identified subject numbers C-1 through C-4.

**Table 2:** Brain areas where significant main effects of group were observed.

Side	Brain area	BA	Cluster size (mm^3^)	MNI coordinates			
				*x*	*y*	*z*	*T*max
PTSD > control							
L	Fusiform gyrus	37	744	−42	−54	−20	6.33
		19		−38	−68	−18	6.25
R	Putamen		200	32	−10	−8	5.48
R	Amygdala			26	−6	−12	4.75
R	Caudate		208	10	−6	22	5.21
				12	−2	14	3.86
L	Caudate		280	−8	4	8	4.85
R	Angular gyrus	19	152	40	−78	44	4.73
L	Thalamus		280	−18	−20	14	4.6
				−12	−26	12	4.45
L	Superior frontal gyrus	8	104	−22	38	48	4.53
R	Inferior frontal gyrus	9	96	60	20	26	4.24
R	Thalamus		104	16	−4	12	4.12
Control > PTSD							
No significant areas of activation							
